# Reframing Eczema: Th2-Skewed Contact Sensitization, Atopy Patch Testing, and Systemic Contact Dermatitis

**DOI:** 10.1007/s11882-026-01260-x

**Published:** 2026-03-13

**Authors:** Sharon E. Jacob, Andrew Scheman, Susan Nedorost

**Affiliations:** 1 Dept of Dermatology, Loma Linda University, Loma Linda, CA USA; 2 VA Loma Linda Health System, Loma Linda, USA; 3 Dept of Medicine-Pediatrics, University of California Riverside, Riverside, CA USA; 4 Dept of Dermatology, Northwestern University Feinberg School of Medicine, Chicago, IL USA; 5 Dept of Dermatology, Case Western Reserve University, Cleveland, OH USA; 6 DOCS Dermatology Columbus, Columbus, OH USA

**Keywords:** Atopic dermatitis, Allergic contact dermatitis, Systemic Contact Dermatitis, Food-triggered Dermatitis, Innate lymphoid cells, Intrinsic Atopic Dermatitis

## Abstract

**Purpose of Review:**

We review allergic contact dermatitis (ACD) with predominant Th2 type cytokine expression in the context of chronic cutaneous inflammation. While more has been written about Th1 skewed ACD due to potent allergens in the setting of healthy skin, this review highlights recognition of Th2 skewed ACD in both atopic dermatitis and systemic contact dermatitis and the role of allergen avoidance as an alternative to systemic therapies.

**Recent Findings:**

Th2 skewed ACD rarely occurs to potent allergens. It more commonly occurs in response to allergens considered “non-sensitizers” in the local lymph node assay. These sensitizers include weaker allergens (e.g. propylene glycol), larger molecules (e.g. food proteins) and commensal micro-organisms. Importantly, group 2 innate lymphoid cells and natural killer T cells may contribute to these cutaneous memory responses without education of Th2 cells in the local lymph node and without downstream antigen-specific IgE. The resulting intrinsic atopic dermatitis may be food-triggered and better diagnosed with atopy patch testing than with tests for antigen specific-IgE used for immediate type hypersensitivity reactions.

**Summary:**

Chronic contact, atopic, and stasis dermatitis all occur in the setting of irritant dermatitis and microbial dysbiosis. Both Th1 and Th2 cytokines mediate ACD, although those cytokines may arise from innate immune pathways and not exclusively from T helper cells educated in the local lymph node. More refinement and use of atopy patch tests to identify less potent allergens and dietary avoidance of patient-specific allergens may reduce the number of patients who require systemic therapy for eczema.

## Introduction

Dermatitis, also known as “eczema,” encompasses a spectrum of pruritic, erythematous, and exudative skin conditions characterized histologically by epidermal spongiosis. These can be divided into “primary” forms—such as atopic dermatitis (intrinsic and extrinsic), contact dermatitis, and stasis dermatitis—and “secondary” mimics like bullous pemphigoid, dermatomyositis, and cutaneous T cell lymphoma [1]. This work focuses on the causes of the primary forms of dermatitis: irritant dermatitis, allergic sensitization, and microbial dysbiosis and the bidirectional interplay between each of these. We offer clinical pearls and diagnostic strategies that support precise diagnosis of triggers as opposed to empiric elimination of multiple potential triggers.

## Irritant Dermatitis Influences Allergic Sensitization

### Acute Irritation

Barrier disruption is the basis of all dermatitis. Potent allergens have irritant properties, and potent allergens sensitize healthy skin resulting in a Th1 skewed allergic contact dermatitis [2]. Acute irritation favors a Th1 skewed response to potent allergens as defined by the local lymph node assay [3]. Potent allergens like poison ivy (Toxicodendron) function as their own irritants [4] and don’t require pre-existing skin disease or injury. Applying neomycin immediately after an abrasion (an acute wound) is an example where an irritant response may occur that results in sensitization.

#### Clinical Pearl

In patients with new-onset dermatitis, it is important to assess for recent injury or exposure to conventional potent allergens (e.g. resins like epoxy or acrylates) that cause a Th1 response without antecedent chronic irritation.

### Chronic Irritation

In contrast, sensitization in the setting of chronic irritation differs from the classical Th1 skewed allergic contact dermatitis, Chronic moderate-severe skin irritation (e.g. from repeated wet/dry cycles due to wet work, or drooling or friction in flexural areas in atopic dermatitis) impairs skin integrity and decreases the likelihood of sensitization response to a potent allergen [5] while at the same time promoting Th2 skewed sensitization towards weak allergens [2, 5], This paradoxical reaction of the skin becoming more reactive to less potent sensitizers like propylene glycol and food proteins [6], is an important consideration, given that these types of allergens are considered ‘non-sensitizers’ in the local lymph node assay [7]. Notably, chronic irritation also promotes the Th2 type cytokines that are produced either by Th2 cells educated in the local lymph nodes in the adaptive response or via innate pathways [8].

As is seen in atopic dermatitis, contact dermatitis and stasis dermatitis, a background of chronic irritant dermatitis driven by repetitive epidermal damage, such as friction and wet/dry cycles, activate keratinocytes to release Th2-skewing cytokines like interleukins (IL-33, IL-25) and thymic stromal lymphopoietin (TSLP) [9]. These “alarmins” instruct the immune system to favor Th2 pathways, laying the groundwork for sensitization to weak allergens—including foods, additives, and commensal organisms.

#### Clinical Pearl

In patients with chronic irritant dermatitis, such as from perspiration and friction in the antecubital and popliteal fossae of children with atopic predisposition, or from drooling in the perioral skin of genetically predisposed infants (both phenotypes characterized as “atopic dermatitis”) assess for exposure to less potent allergens such as lanolin, tocopherol (vitamin E), propylene glycol, and food proteins.

## Sensitization

### Th1 Skewed Sensitization

In classic Th1-type ACD, sensitization begins when low molecular weight haptens bind to self-proteins in the skin—a process known as haptenization. This triggers innate immune activation via Toll-like receptors (e.g. TLR4) and nuclear factor kappa B (NF-κB) leading to the release of type 1 interferon (e.g. IFN-alpha). These signals recruit Langerin+ classic dermal dendritic cells (DCs), which migrate to regional lymph nodes and present antigens to naïve T cells causing a Th1 type response on re-exposure to the sensitizer [10].

### Th2 Skewed sensitization

Keratinocytes, once viewed as passive structural cells, are now recognized as active immunologic instructors. In acute injury, they release IL-1 and activate antigen-presenting cells (APCs) which promotes a Th1-based immune response. In the clinical setting of chronic skin disruption, repair and perturbation—from friction or wet/dry cycles —there is release of alarmins like IL-33 and TSLP, which skews the immune response toward Th2 activation and an enhanced Th2 response [11].

### Role of the Innate immune system in sensitization

Innate lymphoid cells (ILCs**)** reside in the skin and orchestrate immune responses to pathogens and allergens without processing these on antigen receptors. Among them, ILC1s generate interferon gamma and lead to a Th1 response, while activated ILC2s respond to IL-33 and TSLP by producing IL-5 and IL-13, driving Th2-type inflammation and tissue remodeling, which leads to the adaptive clinical response of thickening of the inflamed epidermis and dermal fibrosis i.e. lichenification [12]. ILCs are highly adaptable and can transform their phenotypes in response to different types of external stimulation, such that ILC2 cells can transform to an ILC3 phenotype in response to antigens like yeast [13].

Furthermore, in chronically inflamed skin, ILC2s and natural killer T-cells can generate memory-like responses locally—without lymph node education or IgE production [14]. This is the basis for flare responses in “intrinsic atopic dermatitis”, which means there is no antigen-specific IgE driving the inflammatory cascade. Likewise, contact allergy to large molecular weight allergens (e.g., food proteins) may also trigger systemic flares upon ingestion (systemic contact allergy) without immediate-type allergy via this same mechanism [15]. For this reason, tests for immunoglobulin -mediated hypersensitivity are generally not useful to identify triggers of dermatitis.

#### Clinical Pearl

The absence of antigen-specific IgE or negative prick tests does not exclude Th2-type cytokines as an antigen-driven cause of dermatitis.

Intrinsic AD may involve Th2 memory responses independent of classical pathways involving education in the local lymph node that generate antigen specific IgE. The “Choosing Wisely” high value care campaign cautions not to routinely order prick or RAST testing to evaluate dermatitis [16]. On the other hand, selective patch testing may be of higher value and is outlined in the section on atopy patch testing below.

### Eosinophils: Nomadic Amplifiers of Th2 Inflammation

Eosinophils, recruited via IL-5 and chemokines, migrate from bone marrow to inflamed skin in allergic states. Once in the skin, the eosinophils respond to IL-33 by amplifying IL-5 production and mediating fibrotic tissue damage (lichenification, a hallmark of Th2 inflammation) [17]. Th2 inflammation is promoted by ILC2s, which like eosinophils, express CD80. Notably, ILC2s deficient mice cannot recruit eosinophils or mount a Th2 epithelial response [18].

#### Clinical Pearl

Peripheral eosinophilia is suppressed by systemic steroids. Always assess eosinophil counts when the patient is not taking systemic corticosteroids to avoid false negatives in eosinophilic disorders or drug reactions.

### Sensitization to the Cutaneous Microbiome

*Staphylococcus aureus* heavily colonizes the inflamed skin sites in patients with atopic dermatitis and forms biofilms with fungi, such as Alternaria. This in turn triggers antimicrobial peptides (AMPs) that further damage keratinocytes and perpetuate Th2 signaling [19, 20]. These biofilms formed by *Alternaria* and *Staph aureus* in patients with childhood onset dermatitis promote sensitization [21].

#### Clinical Pearl

A healthy skin barrier resists microbial dysbiosis and sensitization. In patients with new-onset dermatitis due to acute barrier disruption, there is lower probability of microbial dysbiosis than in chronic dermatitis. Conversely, in the setting of chronic dermatitis, allergic sensitization to the elements of the microbiome can occur.

Chronically inflamed skin is prone to microbial dysbiosis, characterized by reduced microbial diversity and increased abundance (e.g. of Staphylococcus *aureus*, *Malassezia*, and *Alternaria*) leading to increased concentration of microbial proteins per unit area which further promotes sensitization.

Contact sensitization to microorganisms, such as Malassezia, can cause a shift toward ILC3-like profiles (producing IL-17 and IL-26) [22] which can present as a sebopsoriasis-like presentation of eczema. Head and upper torso dermatitis in post-pubertal patients often reflects IL-17-mediated sensitization to *Malassezia*. Cytokine profiling of positive patch tests to *Malassezia* yeast showed no IL-4 at either early or late readings [23].

#### Clinical Pearl

Patch tests to confirm *Malassezia* allergy are not commercially available, so this diagnosis is often confirmed by diagnostic and therapeutic challenge with systemic antifungal therapy (e.g., azoles) followed by topical therapy (e.g. ciclopirox) to delay re-colonization.

Occlusion worsens microbial dysbiosis by increasing moisture, warmth and maceration, which further promote growth of micro-organisms. Overuse (more than once a day) of occlusive moisturizers and use of fabrics that do not wick moisture (e.g., polyester or nylon) may also worsen dysbiosis.

#### Clinical Pearl

Optimize use of occlusive moisturizers to a post-bathing regimen once daily [24, 25] and use non-occlusive fabrics like cotton or lyocell in active- wear and nightwear to absorb perspiration and improve air flow for patients with dermatitis [26].

## Elicitation

Once contact sensitization occurs, re-exposure to the sensitizing allergen can trigger memory responses. The allergen activates CD8 + cytotoxic T cells, leading to keratinocyte apoptosis, cytokine and chemokine release, and recruitment of lymphocytes to the skin which in turn cause a clinical flare of dermatitis.

This cascade is amplified by mast cells and neutrophils. However, regulatory T cells (Tregs) can suppress this response—especially in areas like the scalp, which have high Treg density.

### Clinical Pearl

The scalp may remain unaffected in ACD to hair products—even when facial skin flares—due to enrichment of Treg cells in hair follicles [27].

Factors that mediate strength of elicitation have been less studied than those that influence sensitization. For example, it has long been known that UVB exposure at the time of initial antigen exposure promotes tolerance. It is also now known that UVA and UVB exposure can suppress the elicitation response [28].

### Clinical Pearl

From a diagnostic perspective, it is important that patients avoid sun exposure to sites where patch testing will be performed for at least 2–4 weeks prior to testing to prevent false negative patch test results.

In addition, it is notable that one can elicit both Th1 and Th2responses in the same subject at the same time. In patients with atopic dermatitis, the Th2 sensitization tends to be more durable, while the Th1 response is more transient [29].

### Atopy Patch Testing

Atopy patch testing detects less potent, high molecular weight allergens that often have a Th2 skewed response. These reactions peak at about 48 h and are morphologically papular. Conventional patch testing identifies contact allergy to more potent, low molecular weight allergens which provoke more indurated, often vesicular reactions between days 3 and 10 after patch test placement. Failure to appreciate early, papular reactions as positive results to weak antigens results in missed diagnose. Atopy patch testing denotes intent to interpret results at 48–72 h and to assess for number of papules.

As discussed in the section above on eosinophils and ILC2s, the involvement of these cells in dermatitis suggests an interplay between the innate and adaptive immune response that differs from conventional antigen presentation. Of interest, atopy patch tests to aeroallergens in atopic dermatitis patients, but not in healthy controls or respiratory atopy patients (mediated by antigen-specific IgE), show an influx of eosinophils around 24–48 h after applications [30].The term “atopy” is therefore a misnomer for patch testing to weakly potent allergens.

Atopy patch tests are often applied in slightly larger test chambers (e.g. 12 mm) and are removed at 48 h and read at 48–72 h. The morphology of the cutaneous response often demonstrates a papular configuration with the number papules correlating with the strength of the test result.

#### Clinical Pearl

In patients with chronic dermatitis, consider dual patch testing. Conventional tests for potent allergens (read at 96 h–7 days) and atopy patch tests for weak or ingested allergens (read at 48–72 h).

Some allergens that are included on conventional patch test series contain components of foods, cross react with foods, or are used as food additives and as such should be evaluated as if they were applied as an atopy patch test. For this reason, an early reading with a papular response should be considered positive, even if does not persist to 72 h or beyond. Examples of patch tests for allergenic components of foods include balsam of Peru, diallyl disulfide (garlic), metals, and compositae (e.g.: chamomile, ragweed, tansy, feverfew and artichoke) related plants, as well as food additives such as propylene glycol, carmine, vanillin, and benzoic acid. Other allergen sources to be considered for atopy patch tests on a history driven basis include food proteins such as milk, egg, soy, wheat, corn, and oat. These should not be tested in infants prior to ingestion of the food, as this could in theory cause active sensitization before oral tolerance has been established.

As with any allergen that is commonly tested in clinical practice, the likelihood that an individual patient will have a positive test to that allergen is less than 10% (with nickel and Brazilian propolis as possible exceptions). This is true with atopy patch tests as well. Negative tests are particularly helpful when testing to foods and food additives, because they can alleviate the burden of unnecessary dietary avoidance.

#### Clinical Pearl

Ensure complete wash-out of systemic immunosuppressants prior to any conventional (low molecular weight hapten), atopy patch testing or oral provocation testing to prevent false negative results. This requires prior discontinuation for weeks with JAK inhibitors and for months with biologics, although there is not yet consensus on ideal timing [31].

## Food Allergy of the Delayed Type is a Form of Systemic Contact Dermatitis

In approximately 15% of patients with a positive patch test response, ingested allergens can trigger a cutaneous recall response. This usually occurs in patients with early onset flexural dermatitis; ingestion of sensitizing foods or additives triggers systemic flares of eczema, urticaria, or dyshidrotic eruptions. This phenomenon, called systemic contact dermatitis, is mediated by Th2 pathways and may be missed by conventional patch testing with readings only performed after 48 h [15]. Thus, an early and a delayed reading are recommended.

### Clinical Pearl

Systemic contact dermatitis (SCD) without immediate type hypersensitivity is usually triggered by ingestion of weakly potent food allergens. Sensitization occurs in the absence of oral tolerance, and barrier disruption in early life is a key risk factor. Atopy patch testing is the diagnostic test of choice.

If confirmation of SCD is desired via oral food provocation testing, the patient must avoid eating the suspected food(s) for 2 weeks prior to the oral provocation test. Reintroduce suspected foods, one every 4 days, and monitor for eczema flare-ups. This is contra-indicated in a patient with history of anaphylaxis.

As discussed above, in healthy skin, Langerhans cells (LCs) and dermal dendritic cells are the primary antigen presenting cells leading to education of T cells in the local lymph nodes. This is often associated with downstream antigen-specific IgE known as “extrinsic” atopic dermatitis. In contrast, ILC2s (which express CD80) can mediate a memory response in chronically inflamed skin. This response may bypass lymph node education [32] and potentially triggers memory-like responses locally, primarily to less potent allergens. Notably, CD80 polymorphisms have been associated with sensitization to these less potent allergens [33].

Skin resident natural killer T (NKT) cells are innate immune cells with memory functions which ‘home’ to prior areas of damage from prior exposure to an allergen in skin [14]. NKT cells express CXCL4, the cognate ligand of CXCL12. CXCL12 polymorphisms have been associated with irritant hand dermatitis in health care workers [34]. Therefore, it stands to reason that the SCD response following ingestion of a triggering allergen, is in effect a recall of ACD to the same allergen, which was initiated by a primary chronic irritant dermatitis that activated immune memory functions at least in part mediated by ILC2 cells educating NKT cells [33].

### Clinical Pearl

Avoidance diets based on history-driven patch test positive results from specific food and food additive testing may prevent the need for systemic therapy.

## Discussion and Conclusion— Toward Precision Care in Eczema

Dermatitis occurs from barrier dysfunction and immune dysregulation in atopic, contact, and stasis dermatitis. At the etiologic core lies irritant dermatitis, often triggered by friction and wet/dry cycles. In genetically predisposed individuals, chronic irritation activates Th2-skewed immune pathways, sometimes leading to contact sensitization to weak allergens and commensal organisms.

ACD has long been viewed only as a Th1-mediated reactions to potent allergens on healthy skin. Atopic dermatitis, by contrast, was defined as a Th2-driven condition, which was unrelated to contact allergy. This binary view led to misconceptions and a narrowed understanding of the interplay between the adaptive and innate immune response. While intact skin readily responds to high potency allergens, Chronic skin inflammation alters immune responsiveness and enables contact sensitization to weak allergens and commensals via Th2 pathways.

There must be recognition that Th2-skewed contact allergy is not as rare as was previously believed—it is underdiagnosed. Patients with dermatitis may react to food additives, commensals, and weak allergens that evade conventional Th1-based testing. These patients may clinically benefit from avoidance to both systemic and skin contact exposures.

We advocate for:

- Understanding that innate immune pathways allow sensitization to allergens that are non-sensitizers or weakly potent in the local lymph node assay, including foods and commensal organisms.
- Reduced use of occlusive moisturizers and non-breathable fabrics in patients with dermatitis to mitigate microbial dysbiosis.
- Increased study and use of atopy patch testing.
- Recognition of Th2 contact allergy as an early, papular patch test response.
- Precision elimination of identified contact and ingested allergens over empiric elimination strategies.

Adoption of these practices should reduce reliance on costly systemic therapies and their adverse effects.

## Key References

- Sun Z, Kim JH, Kim SH, Kim HR, Zhang K, Pan Y, Ko MK, Kim BM, Chu H, Lee HR, Kim HL, Kim JH, Fu X, Hyun YM, Yun KN, Kim JY, Lee DW, Song SY, Lin CP, Clark RA, Lee KH, Kupper TS, Park CO. Skin resident natural killer T cells participate in cutaneous allergic inflammation in atopic dermatitis. J Allergy Clin Immunol. 2021 May;147(5):1764-1777. 10.1016/j.jaci.2020.11.049.
  - ○ The alarmin TSLP in disrupted skin encourages CXCL12 production by fibroblasts. CXCR4+ natural killer T (NKT) cells are capable of trained memory to antigens and bind to CXCL12 in mouse models and human atopic dermatitis skin. There were many more CXCR4+ NKT cells in skin compared to the regional lymph node.
- Nedorost S, Zhang G, Fekedulegn D, Fluharty K, Wang W, Frye B, Baron E, Zug K, Yucesoy B. Weak Sensitizers May Be Associated with CD80 Polymorphisms: Implications for Systemic Contact Dermatitis. JID Innov. 2025 May 9;5(4):100382. 10.1016/j.xjidi.2025.100382. PMID: 40519868; PMCID: PMC12167014.
  - ○ The CXCL12 polymorphism (rs197452) is associated with both chronic hand dermatitis and sensitization to less potent allergens. CD80 polymorphisms are also associated with sensitization to less potent allergens. Group 2 innate lymphoid cells and eosinophils are CD80+ and suggest an alternative pathway to sensitization outside of the local lymph node that is at least in part mediated by the innate immune system.
- Ahuja K, Issa CJ, Nedorost ST, Lio PA. Is Food-Triggered Atopic Dermatitis a Form of Systemic Contact Dermatitis? Clin Rev Allergy Immunol. 2024 Feb;66(1):1–13. 10.1007/s12016-023-08977-x.
  - ○ Atopy patch tests detect food-triggered atopic dermatitis with greater than 75% sensitivity and specificity compared to oral food challenge. Many of these patients have intrinsic atopic dermatitis, suggesting that sensitization in the local lymph node did not instruct antigen specific IgE or that sensitization occurred outside of the local lymph node in some cases.

## Data Availability

No datasets were generated or analysed during the current study.

## Declarations

### Generated AI

The authors did not use AI for this work; a reviewer disclosed that they used AI to generate suggestions regarding formatting our manuscript.
